# PRIN: a predicted rice interactome network

**DOI:** 10.1186/1471-2105-12-161

**Published:** 2011-05-16

**Authors:** Haibin Gu, Pengcheng Zhu, Yinming Jiao, Yijun Meng, Ming Chen

**Affiliations:** 1 Department of Bioinformatics; State Key Laboratory of Plant Physiology and Biochemistry, College of Life Sciences, Zhejiang University, Hangzhou 310058, China

## Abstract

**Background:**

Protein-protein interactions play a fundamental role in elucidating the molecular mechanisms of biomolecular function, signal transductions and metabolic pathways of living organisms. Although high-throughput technologies such as yeast two-hybrid system and affinity purification followed by mass spectrometry are widely used in model organisms, the progress of protein-protein interactions detection in plants is rather slow. With this motivation, our work presents a computational approach to predict protein-protein interactions in *Oryza sativa*.

**Results:**

To better understand the interactions of proteins in *Oryza sativa*, we have developed PRIN, a Predicted Rice Interactome Network. Protein-protein interaction data of PRIN are based on the interologs of six model organisms where large-scale protein-protein interaction experiments have been applied: yeast (*Saccharomyces cerevisiae*), worm (*Caenorhabditis elegans*), fruit fly (*Drosophila melanogaster*), human (*Homo sapiens*), *Escherichia coli *K12 and *Arabidopsis thaliana*. With certain quality controls, altogether we obtained 76,585 non-redundant rice protein interaction pairs among 5,049 rice proteins. Further analysis showed that the topology properties of predicted rice protein interaction network are more similar to yeast than to the other 5 organisms. This may not be surprising as the interologs based on yeast contribute nearly 74% of total interactions. In addition, GO annotation, subcellular localization information and gene expression data are also mapped to our network for validation. Finally, a user-friendly web interface was developed to offer convenient database search and network visualization.

**Conclusions:**

PRIN is the first well annotated protein interaction database for the important model plant *Oryza sativa*. It has greatly extended the current available protein-protein interaction data of rice with a computational approach, which will certainly provide further insights into rice functional genomics and systems biology.

PRIN is available online at http://bis.zju.edu.cn/prin/.

## Background

Proteins seldom perform their biological function independently. Rather, they collaborate with other biological molecules such as nucleic acids and proteins to accomplish complex biological processes. Protein-protein interactions play fundamental roles in almost all biological processes such as signal transduction, internal equilibrium maintenance and organs formation [1]. Consequently, mapping genome-wide protein-protein interactions has been one of the key tasks of systems biology to understand cellular processes [2].

High-throughput experiments, like Yeast two-hybrid system (Y2H), AP-MS method and Bimolecular fluorescence complementation (BiFC) [1], have been employed widely at genome-scale to construct protein-protein interaction networks of model organisms such as *Saccharomyces cerevisiae*, *Caenorhabditis elegans*, *Drosophila melanogaster*, *Homo sapiens *and *Escherichia coli *K12 [3-11]. But large scale experiments are far from widespread use due to huge financial costs and time consuming experiments. Computational approaches provide a rapid and convenient scan for a preliminary sight before the commencement of comprehensive observations of experimental proteins interaction. They also provide reasonable complements to existing experimental protein interaction networks. With in-depth study of experimental protein interactions, especially the increase of model organisms' protein interaction data generated by large-scale and high-throughput experiments, computational approaches to predict protein-protein interactions in a particular species have been increasingly efficient and effective. Combined with literature extraction of existing protein interactions [12], genomic information, protein structure and annotation information, bioinformatics play an important role in method study of protein-protein interaction prediction, high-quality protein-protein interaction databases establishment, software and webserver development for visualizing protein-protein interaction networks and genome-scale analysis of protein interaction networks [13-18].

Although protein-protein interactions confirmed in the lab are in a low coverage of the whole proteome, including those of model organisms such as human and fruit fly [7-9,11], these findings can offer useful biological information for the prediction of novel protein-protein interactions in a particular species of interest. Machine learning methods like Naïve Bayes [19] and SVM [20] have been used to extract biological information from golden-standard protein interaction data to model classifiers for prediction. Such computational methods depend highly on the reliability of golden-standard data; hence show limitations in organisms that have little existing experimental data for training.

Genome information is another important source for protein-protein interaction prediction. These types of method usually use genome information (gene neighbourhood, gene fusion, domain fusion, gene co-expression, phylogenetic profile, subcellular co-location, domain interaction and GO similarity etc.) to obtain functional dependence between protein pairs [21]. Gene neighbourhood hypothesizes that if proteins in different genomes are corresponding to adjacent genes, these proteins are considered to be functionally related and therefore are more likely to interact with each other [22]. Gene fusion means that if two functionally related proteins in a genome possess homologous similarities, and they can be fused into a compound protein, these two proteins are likely to interact with each other [18]. Phylogenetic profile describes the presence of homologous proteins in a series of species. By clustering phylogenetic profiles, proteins with similar or identical phylogenetic profile patterns are considered to be functionally linked, and they are more likely to interact with each other as well [23]. Early methods usually considered genomic information independently (domain-domain interaction methods used in SynechoNET [24]), but more and more prediction methods combine several or all genome information together to improve the precision of prediction (integrated methods used in AtPID [25,26]).

Methods based on evolutionary information such as correlated mutation, interologs [11,27], correlated evolutionary rate have achieved dramatic improvements in cross-species protein-protein interaction prediction. Evolutionarily conserved protein-protein interaction is based on the theory of evolutionary conservation of protein, which is known as ortholog. The interolog method is mainly dependent on protein ortholog algorithms such as InParanoid [28-30]. Orthologous proteins are used to locate conserved protein-protein interactions among species. It has been proved that many pathways such as GTPase signaling transduction significantly show their evolutionary conservation in different species, especially the pathway motif (patterns that recur within pathways much more often than expected at random), appears in many different pathways [31]. Over the years these prediction methods of protein interactions have been successfully applied in human, yeast, fruit fly and other model species, achieving appreciated results [32,33]. The Online Predicted Human Interaction Database (OPHID [33]) extracts the evolutionarily conserved orthologous protein-protein interactions from *Saccharomyces cerevisiae *and *Drosophila melanogaster *with the interolog method, which is then combined with literature mining data to construct the human protein interaction network. The most important trend of interolog methods is taking both the orthologous information and genomic information into consideration to obtain high quality protein-protein interaction networks, such as the approaches applied in MPID [34] and AthPPI [35].

The complexity of plant materials presents a big obstacle to find analytical protein-protein interactions in plant proteomics research [36]. The genome-scale experimental approach-based plant interactome has not been constructed, only a few protein-protein interaction networks are constructed to address several particular biological questions. *Arabidopsis thaliana *is the only plant species in which a global-goal applicable interactome was computationally constructed [37]. There are several *Arabidopsis thaliana *protein-protein interaction databases with different approaches: AtPIN [38] and AtPID [25,26] with integration approach, AthPPI [35] and Predicted Arabidopsis Interactome [36] with interolog approach, and PAIR [39,40] with machine learning approach. However, publicly available computational protein-protein interaction resource for the model monocotyledon *Oryza sativa *is still lacking.

Rare experimental protein interaction data and low level of genomic annotation information are the two main barriers for computational methods to be widely used for *Oryza sativa*. Machine learning methods such as SVM and Naïve Bayes Network require both high quality golden-standard experimental data and huge genomic annotations. As a result, interolog method combined with limited rice genome information appears to be a realizable way to construct an unprecedented rice protein interaction network. In this study, we attempt to computationally depict a panorama of rice interactome with interolog method, where genome information such as GO annotations, subcellular localization information and gene expression data are utilised to validate the predicted protein interaction network and at the same time, to extract significant biological network properties.

## Results

### Network construction

**Figure 1:**
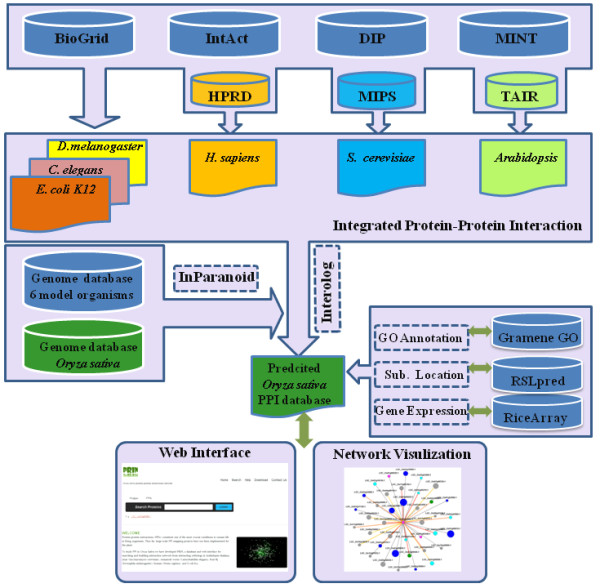
**Multi-species interolog flowchart and PRIN architecture**. Protein-protein interaction data of reference organisms (yeast, human, fruitfly, worm, *E.coli *and *A.thaliana*) are integrated from public databases. The orthologous protein groups between rice and these reference organisms are clustered by InParanoid algorithm. A mapping process, known as interolog, is used to predict protein-protein interactions. Genomic features of rice are additionally mapped to the predicted interactome, RSSGO scores (see Results and Methods), co-localization and co-expression Pearson correlation coefficient scores are calculated for further network validation and database annotation. Finally a well organized web server PRIN is developed to visualize our network and search database.

Our network construction is divided into two main parts: (i) integration of six reference model organism interactomes and (ii) interologs between rice and the reference organisms, as shown in Figure 1.

**Figure 2:**
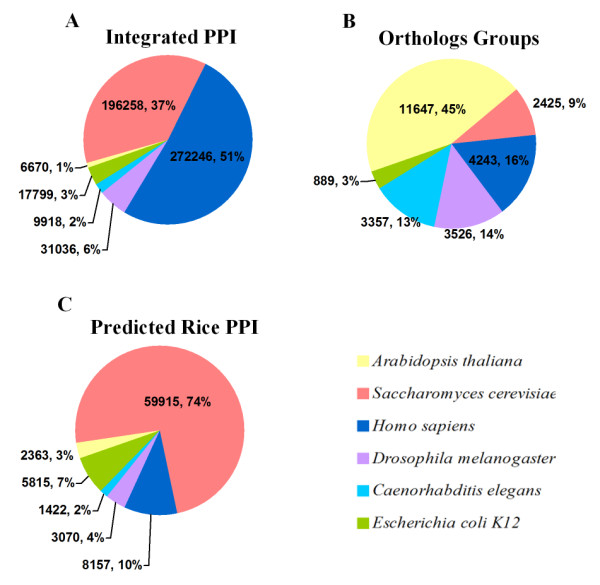
**Multi-species interolog distribution**. Interologs from different species are clustered in different colours. Three kinds of data are counted: A. integrated protein interaction, B. ortholog protein groups and C. predicted rice protein interactions. *Homo sapiens* contributes most protein interactions in our integrated protein-protein interaction database, and *Arabidopsis thaliana* contributes most in InParanoid orthologous protein groups, which shares the highest evolutionary conservation with rice. Nearly 74% of our final predictions are from *Saccharomyces cerevisiae*, which has the highest coverage of its whole genome.

Experimental protein-protein interaction data of model organisms is constantly increasing at a high rate. In order to obtain high coverage and accuracy of our predicted protein-protein interactions, we started with the re-integration of public protein interaction databases and species-specific protein interaction databases. Six model organisms are selected as the reference species for our prediction: *Arabidopsis thaliana*, *Saccharomyces cerevisiae*, *Caenorhabditis elegans*, *Drosophila melanogaster*, *Homo sapiens *and *Escherichia coli *K12, whose experimental interactomes are the most complete and reliable. Among these 6 model organisms, *Arabidopsis thaliana*, a plant species, logically shares the highest evolutionary conservation with rice, while  *Saccharomyces cerevisiae* has the best coverage of its genome. We derived their protein interaction data from public non-species-specific protein-protein interaction databases: BioGrid [41], IntAct [42-44], MINT [45-47] and DIP [48], and additional data is from species-specific databases: MIPS [49] for yeast, HPRD [50,51] for human and TAIR [37] for *Arabidopsis thaliana*. As high-throughput experiments are well-known for their high rate of false positives, there are many noisy records with redundancy and inaccuracy in public protein interaction databases. We either corrected or discarded these errors during the integration process. With their relatively less redundancy and inaccuracy, species-specific databases offer important supplementary data to our integration. We finally integrated 533,927 interactions with 48,152 proteins of the 6 model organisms, which significantly exceeds previous orthologous data used in interolog predictions. As shown in Figure 2, we integrated 6,670 interactions with 3,025 proteins of *Arabidopsis thaliana*, 196,258 interactions with 6,256 proteins of *Saccharomyces cerevisiae*, 272,246 interactions with 22, 986 proteins of *Homo sapiens*, 31,036 interactions with 8,064 proteins of *Drosophila melanogaster*, 9,918 interactions with 4,762 proteins of *Caenorhabditis elegans *and finally, 17,799 interactions with 3,059 proteins of *Escherichia coli *K12 (Additional File 1). InParanoid, as mentioned in background, calculates the ortholog among proteins based on its own algorithm, using the best blast score. We picked InParanoid as the ortholog algorithm because its reliability and availability have been proven among many other interolog methods. InParanoid clusters ortholog pairs with "bootstrap confidence values" and "inparalog scores". "Inparalog scores" reflect the conserved evolutionary distance between an ortholog pair. In order to restrict the sensitivity and definition of our prediction, only ortholog pairs with top "inparalog" (score cut off 1.0) were selected in our interolog method. The distribution of orthologs from 6 model organisms is shown in Figure 2. By mapping the latest version InParanoid7 [29] orthologous to our integrated protein interaction database, we identified 76,585 predicted interactions with 5,049 rice proteins. Among these interactions, 2,891 interactions are found in more than one model organism.

Datasets based on InParanoid7 ortholog pairs without score cut-off were also generated in our study, 1,144,911 protein interactions with 12,709 rice proteins were predicted totally (can be downloaded from our website), which showed high redundancy and low sensitivity based on our examination. As reported by Huang et al. [32], true positive rate of prediction is significantly reduced with the reduction of the InParanoid score cut off, we omitted all this huge amounts of data from reliable rice protein interactions, and score cut off with 1.0 was taken as an internal quality control.

### GO annotations of predicted interactome

**Figure 3:**
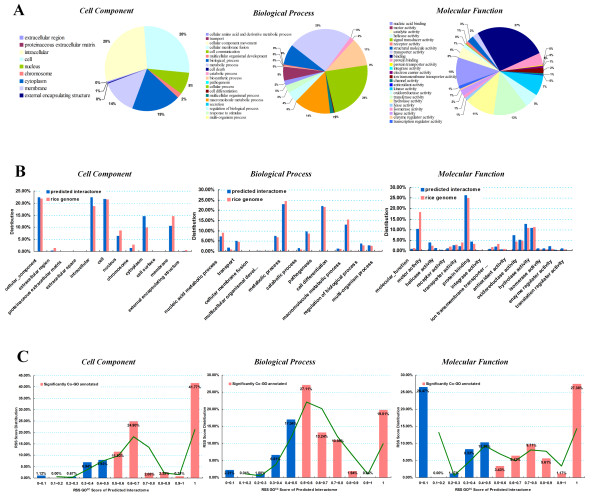
**Gene Ontology annotation analysis**. A. The distribution of GO terms is cataloged based on the UniProtKB-GOA GO Slim. Molecular Function GO terms "binding", Biological process GO terms "cellular amino acid and derivative metabolic process", and Cellular Component GO terms "intracellular" appear most frequently in our calculation. B.GO terms distribution of PRIN (blue bars) shows a high specific similarity towards the GO terms distribution of rice genome (red bars), which shows that PRIN has appreciated coverage over the whole cellular processes. C. RSScc, RSSBP and RSSMF scores (see Methods) are calculated independently except self-interacted protein pairs.

Gene Ontology (GO) is an important bioinformatics tool for genome-scale protein function annotation. GO tries to explain the roles of genes or proteins in eukaryotic cellular process through the establishment of a controlled vocabulary. GO consists of three separate ontologies: cellular component (components of cells or extracellular), molecular function (basic activities of a gene product at the molecular level, such as binding or catalysis) and biological process (collection of molecular events or operation, with a strict definition of the beginning and end). GO uses directed acyclic graph to connect each ontology and renders tree hierarchical relationships between these ontologies. Two proteins involved in the same biological processes have higher possibility to interact than two proteins that are not. Moreover, the more specific a biological process the two proteins are involved in, the likelihood of interaction is higher. Similarly, a more detailed GO annotation will provide a higher chance of interaction. GO mapping can provide an effective measure of the possibility of predicted protein interactions that occur naturally. We mapped the GO annotations derived from Gene Ontology database [52,53] and Gramene [54] to the predicted rice interactome, finally obtaining 4,277 proteins in our network that were highly annotated, with over 84% coverage. We used the well-known GO Slim classification system to measure the distribution of GO terms in our networks. We chose the standard UniProtKB-GOA GO Slim to construct catalogs for GO terms, and GO Slim Viewer provided by AgBase [55] were taken to calculate the distribution of Go terms in our networks. As shown in Figure 3A, proteins with Molecular Function GO annotated "binding" (27%), with Biological process GO annotated "metabolic process" (25%), and with Cellular Component GO annotated "intracellular" (28%) and appear most frequently in our network. Three separate GO term distribution of the proteins in predicted interaction network were compared with that of the whole rice genome. As shown in Figure 3B, the distribution of proteins with Cellular Component terms vested in "cellular component", "intracellular" and "cell" is particularly similar to rice genome (Additional File 2). This high specific similarity also appears in Biological Process terms vested in "metabolic process" and "cell differentiation"; Molecular Function terms vested in "protein binding" and "hydrolase activity". All these terms display the highest distribution in our network. The Pearson correlation coefficient scores for GO term distribution between our network and rice genome were calculated: score for Cellular Component terms was 0.97, sore for Biological Process terms was 0.99 and score for Molecular Function terms was 0.95. These extremely high Person correlation coefficient scores show that tthe proteins in our predicted network exhibit equal distribution against the whole rice genome, rather than restricted to only several certain biological aspects.

We lead Relative specificity similarity (RSS) scores [56-58] into PRIN to evaluate the GO correlation coefficient between two interaction proteins. Relative specificity similarity scores are mainly based on GO term similarity and GO depth (see Methods). Three independent RSS^GO ^scores were calculated separately, RSS^cc ^scores for GO cell component terms, RSS^BP ^scores for GO biological process terms and RSS^MF ^scores for GO molecular function terms. As the RSS^GO ^distribution shown in Figure 3C, RSS^cc ^scores of our network mainly fall within 0.6~1; RSS^BP ^scores mainly fall within 0.4~0.8; RSS^MF ^scores mainly fall within 0.4~0.9. The high proportion of RSS^MF ^score 0~0.1 is mainly due to the imperfection of rice GO molecular function terms. All the proportions of score 1.0 in three kinds RSS^GO ^scores are very high: 41.77% for RSS^cc ^scores, 19.81% for RSS^BP ^scores and 27.38% for RSS^MF ^scores. This shows that two proteins in PRIN share stronger correlation in GO annotation, indicating a higher possibility to interact with each other.

### Subcellular localization of predicted interactome

Subcellular localization is the specific location a protein or gene product exists in where the cell, such as nuclear, cytoplasm or cell membrane. It plays an important role in understanding cellular organs function and compartmentalization characteristics. Proteins have to fulfill the conditions for space identity during interactions; hence interacting proteins tend to possess the same subcellular localization, known as co-localization. Currently there is no specific subcellular localization database for rice. The rare and scattered rice subcellular localization information presents a difficult task for figures collection. To tackle this, we opted for a computational subcellular localization identifier RSLpred [59], which is signally better than another identifier Plant-PLoc [60] because of its rice species-specific characteristic. With the integration of EBI and TIGR rice protein subcellular localization as predicted by RSLpred, we finally obtained 14,308 interactions with subcellular localization information in our predicted interactome, in which 49.1% is co-localized. Four kinds of protein subcellular localizations were catalogued: chloroplast, cytoplasm, mitochondria and nucleus. Nucleus-nucleus co-localization was responsible for the largest share (nearly 44.3%) among all the protein interactions with subcellular localization information; this may not be surprising because 64.2% of proteins having subcellular localization annotations were predicted to nucleus localized by RSLpred.

### Co-expression of predicted interactome

**Figure 4:**
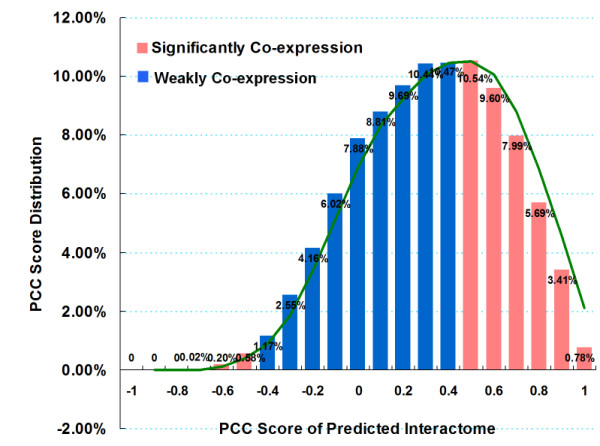
**Co-expression PCC scores distribution**. Pearson Correlation Coefficient score (see methods) is calculated to measure the correlation of two genes expression level of protein pairs in PRIN. Our results show an aggregation in PCC score between 0.3 and 0.8. With cut off 0.5, we finally obtained 28.3% of co-expressed protein pairs in PRIN, which farly exceeds a random level of 2.3% (see Discussion).

If proteins exhibit interaction, there are some quantitative requirements which are closely related to gene expression profile. Using microarray based expression data to predict protein-protein interactions has become a trend in computational systems biology. In protein-protein interaction prediction by gene expression, the most common method is the calculation of the Pearson correlation coefficient between two sets of gene expression data. Many predicted protein interactions based on gene expression profile fall into indirect interactions, also known as function related chains, resulting in high level of false positives in the prediction data. Nevertheless, gene co-expression levels still contain important reference value to protein interactions predicted by interologs. This implies that, no gene expression correlation does not entirely mean that the two proteins do not interact, however, if two proteins gene expression correlate, it will greatly increase the possibility of interaction. Especially, if a protein has a significant inhibition or synergistic effect with another protein as shown by gene expression profile, these two proteins should be strongly considered to interact. In our study, rice co-expression data from Rice Array Database [61] was used to map to our predicted interactome, which is derived from 830 rice Affymetrix microarray data (NCBI GEO AC: GPL2025). The Pearson correlation coefficient score (PCC score shown in Methods) was calculated to measure the correlation of two genes expression. Total of 57,345 of our predicted interactions successfully obtained their PCC score, with a certain Pearson Correlation Coefficient score cut off 0.5; eventually we acquired 16,203 interologs with co-expression relationship. The contribution of PCC score in PRIN is shown in Figure 4. We discovered 2.8% protein pairs with significant inhibition against each other (PCC score < -0.3). However synergy (PCC score > 0) is much more prevail than inhibition (PCC score < 0) in PRIN. Protein pairs in our network mainly fall within 0.3~0.8 section, which shows a significant co-relationship in their gene expression levels.

### Web interface

The web interface of PRIN was developed with JAVA (Struts, iBATIS, Spring frameworks) and was hosted on an Apache web server. The project used MySQL 5.0 as its database management system and Cytoscape Web [62] to visualize the protein-protein interaction network. We provide two ways to access our database: (i) a protein can be queried by its symbol name, Loc number, or UniProt accession in the Protein Search page, then our server will return all the proteins which are predicted to interact with the submission. (ii) If users have a list of proteins and want to know whether they interact between each other, just paste this list of proteins into the submit box of Interaction Search page, then our server will return all the interactions involving these proteins. PRIN provide both graphical results and table results (containing PPI ID, protein ID, Interolog species, co-localization, co-expression PCC score and RSS GO score) for users to get proteins and interactions information. More detail information can be seen through clicking protein ID and interaction ID in the result table.

## Discussion

### Network validation

A small data set of experiments determined rice protein-protein interaction including 406 proteins and 430 interactions is integrated from BIND [63] (Additional File 3), IntAct [44] and PlaPID [64]. Although this experimental interactome is too small a coverage on the rice whole interactome, 95 proteins are also found in our network, which constitute 230 interactions in our network and 66 interactions in the integrated experimental network. Among these 66 interactions, 20 have been determined by experiments, revealing a reasonable sensitive considering the rare and low coverage experimental data.

RSS^GO ^score has been used as a reliable data training method in earlier protein interaction prediction studies [56-58]. In our study, RSS^GO ^score was taken as an inspection method towards our predicted data. We calculated the RSS^GO ^score of protein pairs in our network, both of RSS^CC ^score, RSS^BP ^score and RSS^MF ^score above 0.5 is counted. It was found that 78.9% of them are in a high co-annotated level as RSS^GO ^score cut off 0.5. This indicates that protein pairs in PRIN more likely to participate in the same bioprocess, exhibits similar molecular function and constitute to the same cellular structure, which all leads to a high possibility of protein interaction.

We mapped the gene expression data to the 430 experimental interactions, and successfully found 368 of them with PCC scores, in which 76 pairs having co-expression. Although statistical meaning is obviously lacking due to the limitation of rare experimental data, it significantly indicates that interaction proteins have a tendency to co-express. Protein pairs in our network show an obviously high co-expression rate (28.3%), compared to random pairs derived from global rice genome. We calculated co-expression rate of all gene pairs appeared in the microarray, and only 2.3% of them display co-expression pattern with Pearson correlation coefficient score cut off 0.5. Therefore, the co-expression quality of our predicted network is highly notable.

### Network visualization and topology

**Figure 5:**
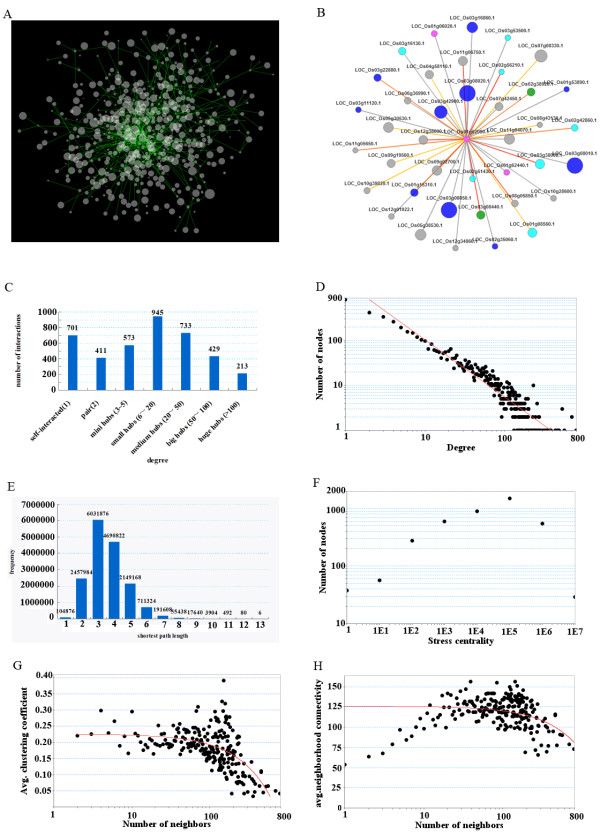
**Network visualization and topology**. A. Part of the core sub network derived from PRIN, and visualized by Cytoscape. B. Insight view of PRIN. Proteins were mapped to 4 subcellular localization: nucleus (blue), mitochondria (cyan), chloroplast (green) and Cytoplasm (pink). Edge colors correspond to co-expression level, and size of nodes corresponds to node degree. C. 7 kinds of hubs were cataloged in our network, partly reference to Jane etal's study [36]. Apart from self-interacted proteins, proteins in PRIN mainly fall into mini hubs, small hubs and medium hubs. D. The shortest path length of PRIN has an aggregation between 2 and 4, showing significant small world properties. E. The node degree distribution of our network shows the scale-free network topological property. F. The high clustering coefficient value indicates that rice protein-protein interaction is highly modular. G. The stress centrality distribution of PRIN implies a strong fault tolerance. H. The first increased followed by a decrease trend in the neighborhood connectivity distribution suggests that it is more prevail that proteins in high degrees interact with low degree proteins in PRIN.

By loading the predicted rice protein-protein interaction data to Cytoscape [62], we obtained visualization of the whole network. We used Cytoscape plug-in NetworkAnalyzer [65] to get topological properties of our network (Additional File 4). An inner-interactome of PRIN is partially shown in Figure 5A. This type of inner-interactome, also found in *Arabidopsis thaliana* and yeast, implies that little proteins interact individually in the interactome [36]. An insightful view of this core interactome is presented in Figure 5B with nodes degree information, subcellular localization and co-expression level.

We made a comparison of network topology properties between the rice interactome and other six species (Table 1). A connected component value of 102 suggests that *Oryza sativa *has similar interactome connectivity with *Caenorhabditis elegans*, stronger interactome connectivity than *Arabidopsis thaliana* and *Homo sapiens*, and weaker than *Saccharomyces cerevisiae*, *Drosophila melanogaster*, *Escherichia coli* K12. Proteins in our predicted interactome possess an average of 29 neighbours, which is closed to *Homo sapiens*, less than *Saccharomyces cerevisiae*, more than *Arabidopsis thaliana, Drosophila melanogaster, Caenorhabditis elegans *and *Escherichia coli *K12. This finding may not be surprising since yeast and human are the major interologs source to our data. It has been shown in yeast and human that the average number of neighbours will increase with upgrading of interactome coverage, which indirectly implies a relatively good coverage on our predicted interactome.

**Table 1:** The comparison of interactome topology between rice and model organisms

species	average degree	connected component value	self-interaction ratio
*Oryza sativa*	29	102	2.8%
*Arabidopsis thaliana*	4	152	5.5%
*Saccharomyces cerevisiae*	61	2	0.8%
*Homo sapiens*	23	157	1.6%
*Drosophila melanogaster*	7	55	0.9%
*Caenorhabditis elegans*	4	105	2.1%
*Escherichia coli *K12	11	9	2.3%

The node degree of an interactome shows the number of edges a protein is linked to, where self-interaction is also counted. The node degree distribution of our network shows the scale-free network topologies of rice protein-protein interaction network. Apart from self-interacted proteins, proteins in PRIN mainly fall within 2-10 degree hubs, as shown in Figure 5C. We constructed hub catalog by partly reference to Jane's studies [36]. As shown in Figure 5D, the node degree distribution of our network shows the scale-free network topological property. Most proteins in scale-free networks have low interactions, and a few proteins have high network connection degrees, which are called distribution node proteins. Scale-free protein interaction network is subject to distribution node proteins, and it has high tolerance to sudden environment pressure.

The shortest path length distribution, as presented in Figure 5E, demonstrates the significant small world property of our predicted rice interactome. The small world properties imply a strong fault-tolerance to our network, as well as to real protein-protein interaction networks [66]. The Small world property shows good fault tolerance and stability of our network. When expressions of a few proteins are suppressed under environment pressure, biological pathways will not be ended but can be completed by other alternative proteins. In such small world networks, information transmission speed is very fast, corresponding with rapid changes of environment pressure. The stress centrality counts how many times a protein being passed through by a shortest path. If a protein is passed by a high amount of shortest paths, it experiences higher stress, suggesting that it has more important biological function [65]. The stress centrality distribution is shown in Figure 5F.

The clustering coefficient is a ratio N/M, where N is given as the number of edges between the neighbours of a protein, and M is given as the maximum number of edges that could possibly exist between the neighbours of a protein. It is calculated as *Cn *= 2*e*_*n*_/(*k*_*n *_(*k*_*n*_-1)), where *k*_*n *_is the number of neighbours of n and *en *is the number of connected pairs between all neighbours of protein n [65]. The average of the clustering coefficients of proteins in different degree is shown in Figure 5G. The high clustering coefficient value suggests that protein-protein interaction in rice is highly modular, and cellular function in real PPI network is likely to be implemented in a highly modular approach. Research in metabolic networks using the average clustering coefficient distribution has shown the modular tendency in metabolic networks [67]. Therefore, clustering coefficient is a very useful methodology to identify functional modular in rice protein-protein interaction network.

The neighbourhood connectivity of a protein, defined by NetworkAnalyzer, is the average interaction numbers of all neighbours of this protein. As shown in Figure 5H, the neighbourhood connectivity of PRIN first increased followed by decreased. This indicates that proteins in low degrees (<30) tend to interact with those of proteins in low degrees, but in high degrees field (>30), it is more prevail that proteins in high degrees interact with those of low degree proteins in PRIN [68].

## Conclusions

PRIN is based on a sophisticated computational method known as interologs, combined with the genomic features of rice. There are certain inner quality controls in our network construction: the huge amount of integrated model organisms' protein-protein interactions, manual proofreading mismatch IDs in database integration, restricted orthologous data with top InParanoid score and manual verification of the resulting network. Genomic feature of rice, such as GO annotations, subcellular location and gene expression data, is mapped to PRIN in order to validate our network and obtain biologically significant results as well. Finally we acquired 76,585 desirable interactions among 5,049 proteins (Additional File 5). According to the comparisons with small experiment interactome and random interactome, PRIN shows satisfactory tendency in co-GO annotation, co-localization and co-expression, making it reliable for perspective studies in rice functional biology and systems biology. A well-organized web interface has been developed for network visualization and database search, which will be updated weekly for new interologs detection. It is publicly available at http://bis.zju.edu.cn/prin/. We have found many conserved basic metabolic pathways among species through the interolog process and most excitingly new protein complexes join known pathways. Pathways expansion, metabolic module detection, and protein complex functional annotation based on PRIN will be the most important features for our further comprehensive genomic functional determination in PRIN.

## Methods

### Interologs

Our prediction is based on existing methods known as interolog. Interolog method is based on a simple logical principle: if 'protein A' and 'protein B' in a specific species are orthologous with 'protein A_1_' and 'protein B_1_' respectively in another species, and the interaction between 'protein A_1_' and 'protein B_1_' has already been verified experimentally in the reference species, 'protein A' and 'protein B' would then be predicted to interact with each other. If interolog of protein A and protein B is found in more than one species, the reliability of their interaction is increased. Interolog method based on multi-species considers evolution conservation between protein interaction pairs, therefore naturally possesses better sensitivity in cross species prediction.

Integration of six model organism interactomes is based on our own integration methods. High-throughput experiments determined and literatures derived protein-protein interaction data of 6 model organisms were gained from public protein-protein interaction databases: BioGrid, IntAct, DIP and MINT. Additionally, species-specific protein-protein interaction databases such as HPRD for human, MIPS for yeast, and TAIR for Arabidopsis were also utilised, providing a significant number of high-quality protein-protein interaction data. An ID dictionary was created to provide cross-database ID mapping, which is based on Biomart, PIR ID mapping service, Uniprot ID mapping service, documents from Swissprot and script extraction from Uniprot XML files. ID mismatching and multi-matching were manually corrected in our integration, and ID in old version was merged into new version or deleted. The ortholog data were gained from InParanoid database between rice and 6 model organisms independently. InParanoid compared all the protein sequence pairs in a species through InParanoid's own algorithm, which is based on blast calculation but not simply the best blast score. Protein with the highest similarity is selected as a candidate protein, ensuring that there is no other protein and its candidate protein has a higher similarity. All orthologous proteins in two species were obtained through these screening methods [28-30]. Only the top pairs clustered by InParanoid core cut-off 1.0 were selected, exerting certain controls on false positive rate of the data. Some orthologs with low score that produce correct interactions are more likely to be false positives. The ortholog data is next mapped to integrated interactome, known as interologs. We finally predicted 76,585 rice protein-protein interactions among 5,049 proteins, with 2,363 interactions from *Arabidopsis thaliana*, 59,915 interactions from *Saccharomyces cerevisiae*, 5,815 interactions from *Escherichia coli *K12, 1,422 interactions from *Caenorhabditis elegans*, 3,070 interactions from *Drosophila melanogaster *and 8,157 interactions from *Homo sapiens*.

### GO annotation

Three independent Gene Ontologies (biological process, molecular function and cellular component) for proteins in PRIN were obtained from the Gene Ontology database [53] and Gramene database [54]. Term description was obtained from Gene Ontology database for network clustering. GO clustering is based on existing methods known as GO slim. UniProtKB-GOA GO Slim [69] was chosen to construct catalog for GO terms, and GO Slim Viewer provided by AgBase [55] was taken to calculate the distribution of GO terms in our networks. Relative specificity similarity (RSS) score of protein pairs in PRIN based on GO annotation were calculated to evaluate of the reliability of the predicted rice protein-protein interaction. We applied tools provided by SPIDer [58] to calculate the RSS^GO ^score between two given GO terms. RSS score is based on existing methods presented by Wu [57]. RSS score can be defined as:

where, α measures specificity between two GO terms (term *i *and term *j*) of a given protein, protein A, and α can be defined as:

where, β measures how relatively general term i and term j are in the GO and β can be defined as:

where, U = {all leaf nodes descending from term i} and V = {all leaf nodes descending from term j}, ɣ measures the local distance between two terms relative to the given protein, and ɣ can be defined as:

And to a given interacted protein pair, protein A and protein B, terms(A) and terms(B) are all the GO terms corresponding to protein A and B. RSS^GO^(A, B) is defined as the correlation strength between A and B [56-58]:

Three independent RSS^GO ^scores were given. With a certain cut off of 0.8, larger RSS^BP ^score indicates that two proteins having stronger correlation in biological processes; larger RSS^CC ^score indicates that two proteins having higher similarity of cell components characteristics; a larger RSS^MF ^score indicates that two proteins are more similar in molecular functions.

### Subcellular localization

Rice subcellular localization data was obtained from the prediction of RSLpred. RSLpred is an integrated prediction server for rice subcellular localization based on four kinds of SVM modules: amino acid composition, dipeptide composition, pseudo amino acid (pseAA) composition and evolutionary information of PSI-Blast. RSLpred classified rice proteins into four 4 subcellular locations: chloroplast, cytoplasm, mitochondria and nucleus. The complete rice proteome of EBI and TIGR were predicted by RSLpred with a faster and traditional amino acid composition based module, and these two files were downloaded and combined to get the maximum coverage over our protein interaction network. Considering the transport mechanism of proteins, we did not adopt the winner-takes-all approach used in earlier studies [36], and all of subcellular localization sources predicted by RSLpred for a single protein were taken into annotation. If one of the localizations of a multi-localized protein were the same with its interaction partner, these two proteins are considered co-localized.

### Co-expression

The Pearson correlation coefficients of two rice genes were obtained from the RiceArray Database [61] calculation based on rice gene expression data in 830 rice Affymetrix microarray data (NCBI GEO AC: GPL2025). Since there were only 34,016 out of 37,993 rice genes (which have Affymetrix probeset matched) with a unique match in Affymetrix probeset, 35% of protein pairs in our predicted rice interactome successfully mapped to co-expression Pearson Correlation Coefficient score. If gene A and gene B are the given two genes, X_i _and Y_i _are the gene expression level of A and B in time *i*, the Pearson Correlation Coefficient score (ɣ) can be given as follows:

where,  mean the average gene expression amount during time *m*, *σx *and *σy *means the standard deviation of gene expression amount during time m. The value of ɣ drops into -1 ~ 1, and -1 means gene expression patterns of A and B are opposite (a expression increased, the other down); 1means that gene expression patterns of A and B are consistent (a expression increased, the other up); 0 means that gene expression patterns of A and B are without any contact. Since interacting proteins may be mutually reinforcing (corresponding to ɣ > 0), may also be inhibited each other (corresponding to ɣ <0), so we use the absolute value of ɣ as a co-expression property between a predicted protein interaction.

## Authors' contributions

MC conceived this study, PCZ designed the method of this study and analyzed the result. HBG processed the data and constructed the database and web interface. YMJ tested the web server. The manuscript were written by PCZ, reviewed and revised by HBG, YJM and MC. All authors read and approved the final manuscript.

## Supplementary Material

Additional file 1 **Statistics of interolog**. This file contains a statistics of interolog data shown in Figure 2. Data resource from protein-protein interaction databases for 6 organisms is listed in table. Click here for file

Additional file 2 **Statistics of GOA GO Slim**. This file contains a statistics of GO terms distribution based on standard UniProtKB-GOA GO Slim shown in Figure 3A and Figure 3B. Three kinds of GO terms(Cell Component, Biological Process and Molecular Function) are listed separately in table. Click here for file

Additional file 3 **430 experiments detected rice protein interactions**. This file contains 430 rice protein-protein interactions, which are integrated from BIND, IntAct, and PlaPID. Proteins are listed in RGAP locus id pairs with their RSS^GO ^score and co-expression PCC score. Click here for file

Additional file 4 **Topological statistics of PRIN**. This file contains detail data resource shown in Figure 5. Topological statistics in hubs distribution, node degree distribution, shortest path length distribution, stress centrality distribution, average clustering coefficient distribution and average neighbourhood connectivity distribution are calculated in table. Click here for file

Additional file 5 **76,585 predicted rice protein interactions in PRIN**. This file contains 76,585 rice protein-protein interactions predicted by our interolog method with high confidence. Protein description, RSS^GO ^score, co-expression PCC score, co-localization level and interolog species are also involved in this file. Click here for file
